# Injuries and Persistent Pain in Elite Adolescent Archery Athletes: A Cross-Sectional Epidemiological Study

**DOI:** 10.3390/sports12040101

**Published:** 2024-04-02

**Authors:** Nikolaos Vasilis, Athanasios Kyriakides, George Vasilopoulos, Maria Chatzitimotheou, Grigorios Gonidakis, Athanasios Kotsakis, Eleftherios Paraskevopoulos, Eleni Kapreli

**Affiliations:** 1 Go Physio Laboratory, Sports Medicine & Rehabilitation Centre, 10675 Athens, Greece; nikosvasilis.uth@gmail.com (N.V.);; 2 Clinical Exercise Physiology and Rehabilitation Research Laboratory, Physiotherapy Department, University of Thessaly, 35132 Lamia, Greecemaria_xatz96@hotmail.com (M.C.);; 3 Physical Medicine & Rehabilitation Department, Mediterraneo Hospital, 10675 Athens, Greece; 4 Physiotherapy Department, University of Peloponnese, 23100 Sparti, Greece; elparaskevop@uniwa.gr; 5 Physiotherapy Department, University of West Attica, 12243 Athens, Greece

**Keywords:** archery, adolescents, shoulder, injuries, persistent pain, training factors

## Abstract

This cross-sectional epidemiological study aimed to evaluate the prevalence of injuries among young archers engaged in high-intensity training during the European Youth Championship. A total of 200 participants (104 males/96 females) from 34 countries were included, with a mean age of 16.9 years and average competitive experience of 6.5 years. Structured questionnaires, administered by four physiotherapist interviewers, gathered comprehensive data. Results revealed that 43.5% of participants experienced shoulder pain during training, highlighting the vulnerability of upper limbs in archers. Additionally, 30% required medications to facilitate training, underscoring the impact of injuries on continued participation. Physiotherapy was utilized by 52.3% of participants, emphasizing the need for therapeutic intervention. Furthermore, 31.8% had to cease training due to injuries, indicating a substantial hindrance to athletic progression. The mean pain duration was 3.9 months, with an average intensity of 5.94, and 8% exhibited symptoms of central sensitization. In conclusion, this study demonstrates a noteworthy prevalence of injuries, particularly in the upper limbs, among young archers undergoing intensive training. The findings underscore the importance of targeted injury prevention strategies and comprehensive rehabilitation approaches to ensure the well-being and sustained participation of young athletes in competitive archery.

## 1. Introduction

The participation of adolescents in competitive sports is increasingly popular and widespread internationally, providing a wide range of health-enhancing benefits [1,2,3]. To specialize and be competitive in a particular sport, several hours of highly intensive training are required weekly [4]. However, the physical requirements to achieve the expected results are not always met, and there is a recognized potential risk of experiencing a variety of acute and overuse injuries [5,6]. This is more evident when comparing the physical demands, injury risks, and training characteristics in archery and other non-contact sports (Appendix A). Such injuries may have significant negative physical and/or psychological effects on the motivation of the young athlete to remain interested in continuing practice and skill development, with dropout being more likely to occur [5,7,8].

Archery is a non-contact sport that involves shooting arrows at a target from set distances. It first appeared in the Olympic games in 1900 and, four years later, became one of the first sports to include athletes from both genders [9]. Archery is considered an ideal sport for children, as, in addition to positive effects on body balance and posture, it enhances their focus, determination, and goal-setting skills, and builds confidence [10,11]. However, competitive archery is a high-demand sport that requires dedication and an intense training regimen. It necessitates strength and stamina, as well as high technique, concentration, and balance [12,13]. All these factors play a significant role in performance. The mental state of the athlete, such as intrinsic motivation and self-perception, further affects performance [8]. 

Young competitive athletes, particularly those in sports like archery, often face significant mental health challenges amidst the pressures of intense training regimens and competitive environments [8]. The pursuit of perfection, coupled with the fear of failure, can lead to heightened levels of stress, anxiety, and even depression [8]. The relentless demands for performance excellence, coupled with the need to balance training commitments with academic or personal responsibilities, can result in feelings of overwhelm and burnout [3,4]. Moreover, the inherent solitary nature of individual sports like archery can exacerbate feelings of isolation and loneliness, further impacting mental well-being [8]. Coping with injuries, setbacks, and the constant pressure to meet expectations can also take a toll on self-esteem and confidence [5]. 

From a biomechanical perspective, the repetitive and asymmetric movements involved in drawing and releasing the bow are identified as the primary causative mechanism for injuries related to archery, particularly affecting the upper limbs. The shoulder area is the most commonly impacted, followed by the elbow and, to a lesser extent, the wrist. Additionally, injuries to the spine and peripheral nervous system have been documented in archery practitioners [14,15,16,17,18,19,20,21]. Despite these findings, there is limited evidence available concerning younger athletes engaged in archery, particularly those participating at a high competitive level. Additionally, there is a lack of documented prevention programs in the literature aimed at reducing injuries among young competitive athletes participating in sports such as archery.

The occurrence of injuries across multiple anatomical areas in this population emphasizes the strain imposed on specific anatomical structures due to the nature of archery movements. The repetitive nature of drawing and releasing the bow can lead to overuse injuries, with the upper limbs bearing the brunt of the stress [22]. Identifying the prevalence of overuse injuries in this population is essential for developing targeted injury prevention strategies and promoting the long-term well-being of athletes engaged in archery, especially among younger athletes participating at competitive levels where the physical demands are heightened. This proactive approach not only safeguards the immediate health of athletes but also fosters sustainable and enduring participation in archery, supporting the overall development and longevity of their athletic pursuits.

Given the intensive training regimens of competitive archers, concerns have been raised regarding the long-term health consequences affecting the young population. Overuse injuries may lead to incomplete recovery and residual symptoms, limiting the ability to engage in pain-free sport participation. The presence of ongoing pain can disrupt motor behavior and alter control of movements during athletic activities, significantly affecting skill and technique [22]. Furthermore, the persistence of ongoing pain can have profound effects on the function of neurons and circuits within nociceptive pathways. This is attributed to increases in membrane excitability and synaptic efficacy, coupled with diminished inhibition that leads to central sensitization [23,24]. Understanding these neuroplastic changes is essential not only for comprehending the physiological basis of ongoing pain but also for developing targeted therapeutic interventions aimed at modulating these nociceptive pathways. Such insights are particularly valuable in the context of injuries related to archery, where the presence of ongoing pain can significantly impact an athlete’s performance, motor behavior, and overall well-being. 

Therefore, the purpose of this study is to add information on the epidemiology of competitive archery injuries in the young population, taking into consideration training characteristics.

## 2. Materials and Methods

The present study was conducted at the Panpeloponnisiako stadium in the city of Patras, Greece, during the European Youth Archery Championship. The inclusion criteria comprised boys and girls aged 13–20 actively participating in the event and possessing the ability to comprehend instructions in either Greek or English. The research employed a structured questionnaire encompassing four distinct sections with specific inquiries.

### 2.1. Instruments/Measurement

The initial section, labeled “personal data of the athlete”, gathered demographic information from each participant. The second section, named “training/bow characteristics”, delved into aspects related to dexterity, dominant arm, and sport participation details. This included data on years of archery experience, practice and game participation hours per week, nutritional habits, and pre-season testing. The third section, titled “pain due to training”, specifically targeted individuals who had encountered archery-related injuries during their active participation, covering aspects such as the localization, mechanism, and type of the pain, as well as therapeutic intervention. The fourth section, designated “general health condition”, explored the presence of any concurrent health issues or prior therapeutic interventions. Confidentiality was rigorously maintained, and all collected information was anonymized in our medical database at the conclusion of the competition. The questionnaire is available in Supplementary Materials.

In this study, the Central Sensitization Inventory (CSI) played a crucial role in identifying young archers exhibiting central sensitization (CS). The CSI has been demonstrated to be valid as a self-report instrument for screening individuals with central sensitization [23,24]. Part A of the CSI comprises 25 items, each rated on a 5-point scale, ranging from 0 (“never”) to 4 (“always”), resulting in a total score range of 0–100. This section is designed to measure symptoms associated with central sensitization. A cutoff score of 40 out of 100 was established to characterize individuals diagnosed with CS. Both the English and Greek versions of the CSI were utilized in this study [25], ensuring comprehensive coverage of the diverse participant population. The incorporation of the CSI strengthens the study’s ability to identify and understand CS among young archers, contributing valuable insights to the overall research findings.

**Figure 1:**
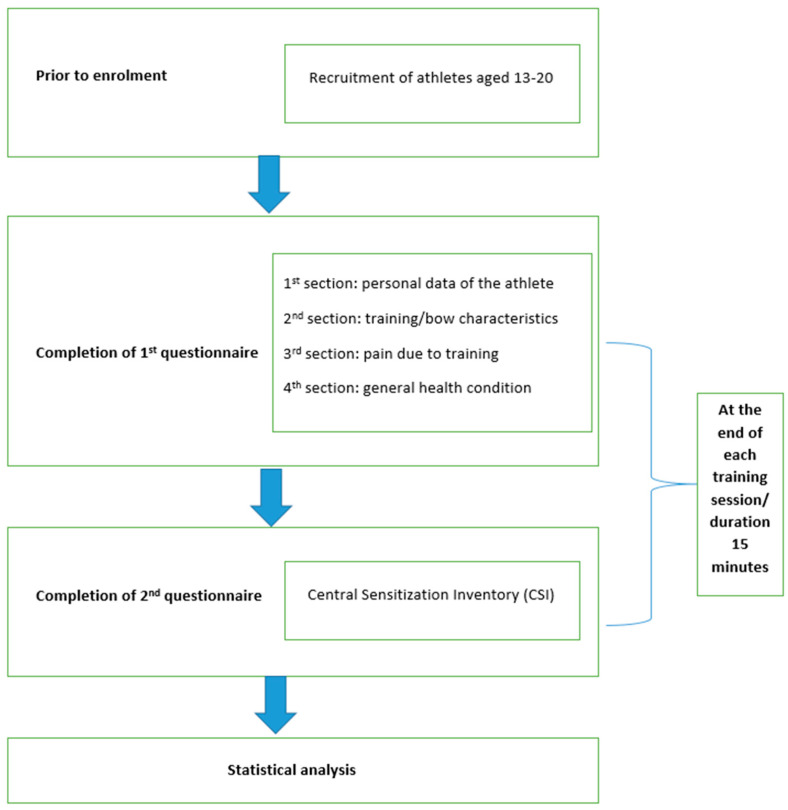
Schematic representation of the experimental design.

The study received approval from the ethics committee of the Physiotherapy Department at the University of Thessaly (Protocol Number: 118/02-10-2018). Prior to commencing the proceedings, athletes were informed through the Candidate Volunteer Information Form from the World Archery Federation that had approved the current study. The form contained detailed information about the study’s content and objectives. Athletes and their guardians (national teams’ representatives) willingly agreed to participate in the research after being fully informed, given their written consent. Following their consent, volunteers proceeded to the designated area set up inside the stadium to complete the questionnaires. The questionnaires were filled out on a daily basis, at the end of their training sessions and before games, with an approximate duration of 15 min. To ensure clarity and prevent misinterpretation of the questions, a team consisting of a physiatrist, two physiotherapists, and two undergraduate physiotherapy students was present to provide oral explanations as needed (Figure 1). 

### 2.2. Statistical Analysis

The statistical procedures were performed using SPSS^®^ 22 (IBM^®^, New York, NY, USA). Statistical significance was defined as *p* < 0.05. Descriptive statistics, including means, standard deviations, frequencies, and proportions, were employed to compare various variables. The chi-squared test was utilized for the comparison of proportions between genders for categorical data, and the independent samples *t*-test was used for variables measured on a continuous scale [26,27]. A Pearson’s product-moment correlation was run to assess the relationship between CSI score and Weekly Training Frequency, Training Duration Per Session, Number of Competitions per year and Competition Duration per Match. 

## 3. Results

This study involved a diverse cohort of 200 elite archers representing 34 countries, comprising 52% males (*n* = 104) and 48% females (*n* = 96). Descriptive characteristics, including age, height, weight, and BMI, were meticulously recorded and analyzed (Table 1). Apart from the anticipated physical traits among the athletes in this group (height, weight and BMI), the statistical analysis revealed no significant differences between genders in the rest of the parameters measured, ensuring a balanced representation of both male and female athletes in the study.

### 3.1. Training Characteristics

Table 1 further provides a comprehensive overview of the training characteristics within the sample. Across genders, no statistically significant differences were observed in various training parameters. A noteworthy observation was that a majority of athletes had accumulated over six years of practice, showcasing a high level of experience within the cohort. The prevalent use of recurve bows among archers was a consistent trend.

Detailed insights into the training regimen were obtained, revealing the mean bow weights for female and male archers (15.44 lb and 16.99 lb, respectively). Training durations averaged 15.44 h per week for females and 14.38 h per week for males. The mean number of arrows shot per training session was 160.36 for females and 165.1 for males. Overall workout intensity, calculated as the product of arrows and drawing weight, demonstrated slightly higher values for males (1271.27 kg) compared to females (1122.52 kg).

The mean bow weight was 15.44 lb (7 kg) for girls and 16.99 lb (7.7 kg) for boys. Female archers reported a mean training duration of 15.44 h per week, while males reported 14.38 h per week. The mean number of arrows shot per training session was 160.36 for girls and 165.1 for boys.

### 3.2. Competition Participation

The study delved into the competitive aspect, indicating that female archers participated in an average of 11.94 competitions per year, whereas their male counterparts engaged in 10.26 competitions annually. Mean competition durations were recorded to be 86.54 min for females and 118.72 min for males, highlighting potential gender-specific variations in competition dynamics.

### 3.3. Training Components and Practices

An intriguing finding emerged in the analysis of training components, revealing a significant discrepancy in emphasis between lower and upper body training. Approximately 51% of the sample neglected lower body training, emphasizing the need for a more balanced approach. Upper limb training primarily focused on strength exercises for both dominant and non-dominant arms.

While stretching before and after training was widely adopted, only half of the archers considered any form of aerobic training important. Notably, relaxation, concentration techniques, and breathing exercises were frequently incorporated into training programs, with a slight predominance observed in males. A mere 55% of the sample reported undergoing an annual physical assessment, and only 32% adhered to a special diet, suggesting potential areas for improvement in overall athlete well-being and performance optimization.

### 3.4. Pain and Injury Analysis

The study identified that 101 participants experienced persistent pain lasting more than three months. Table 2 reveals that 8% of athletes exhibited central sensitization symptoms, providing insights into the complexity of pain experiences in elite archers. An encouraging aspect was the proactive communication of pain or discomfort to coaches, with 83% of archers mentioning their willingness to inform their coaches.

Surprisingly, 31% of archers acknowledged resorting to medications for managing persistent pain, despite reported negative effects (50%) or mild effects (30.77%) for some. An in-depth analysis of pain distribution indicated that 165 athletes reported pain in at least one body area, with 46 experiencing pain in two areas and 5 in three areas. Shoulder joint pain was prevalent in nearly half of the participants (49.1%), followed by spinal pain, with a higher incidence in the lower back compared to the thoracic and cervical spine.

**Figure 2:**
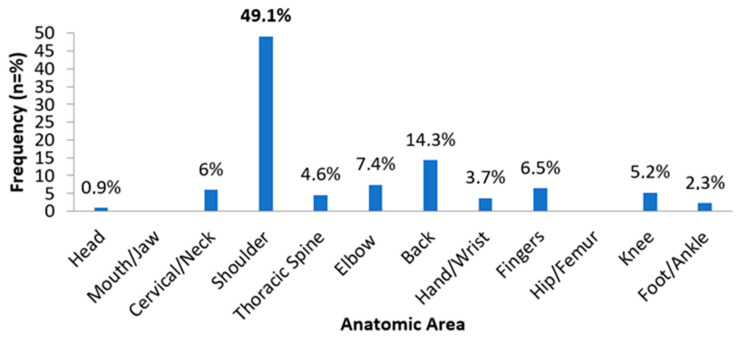
Frequency of injuries for each anatomic area.

Less commonly reported were injuries to the elbow, finger, and knee, which were nonetheless identified as areas of pain interest for these athletes. Hand/wrist and foot/ankle areas were the least reported in terms of pain, as visually represented in Figure 2.

Upon investigating potential correlations between CSI scores and variables such as weekly training frequency, training duration per session, number of competitions per year, and competition duration per match, no statistically significant relationship was observed in the results (Table 3).

## 4. Discussion

Recent literature highlights the potential hazards of severe overuse injuries associated with early sports specialization [6]. Despite archery being deemed a safe, non-contact sport, a number of acute and overuse injuries have been documented, attributed to factors such as training frequency (days per week), intensity (number of arrows shot), and duration, coupled with inadequate body strength and incorrect technique [28,29,30]. In our study, participants engaged in an average of five daily training sessions per week, with a mean session duration of approximately 3 h and an average of 163 arrows shot per session. The yearly involvement encompassed 11 events with a mean participation time of 129 min. Disturbingly, 50.5% of respondents reported long-term pain related to training, and 45% faced challenges in executing proper techniques due to varying degrees of pain. Encouragingly, the majority of our participants appeared proactive in communicating pain-related concerns to their coaches, despite potential shortcomings in the training of youth sports coaches in managing adolescent athletes [31].

Consistent with findings in adult archers, our study indicated that the shoulder joint was the most frequently affected area, with over half of the participants reporting shoulder pain [19,20]. This aligns with existing literature, as the force required to draw the string back to the full drawing position, termed “bow weight”, exerts significant load on the shoulder joint during the shot routine [29]. Moreover, the drawing arm must support this position for several seconds while the archer aims and subsequently releases the string [29].

Our findings also highlight that young archers predominantly focus on upper body strengthening, especially the arm responsible for drawing the string back. This emphasis on strengthening the muscles around the drawing arm aligns with Hidayat’s (2014) recommendation, suggesting that such strengthening could alleviate the loads sustained by the shoulder joint. However, it is crucial to note that strength alone is not sufficient; proper alignment and full-body kinetics are equally essential. Alignment in archery, defined by the formation of a triangle with the shoulders and arms at full drawing position, is critical. Supporting as much of the bow’s draw weight through bone structure rather than muscles is emphasized during the shot routine. Improper alignment, such as setting the front shoulder too high, can weaken the archer’s position and shorten the draw length, posing common challenges for novice archers. Therefore, achieving proper alignment not only enhances performance and reduces the risk of shoulder injuries but is also vital for the athlete’s shooting session longevity and overall career sustainability [32,33].

Moreover, body stability plays a crucial role in shot accuracy, particularly during string release [34,35]. Sustaining a proper standing posture for an extended period enhances shot consistency [36]. Achieving an accurate standing posture necessitates core and lower limb strengthening to improve balance and body stability. Notably, our results indicate significant neglect of lower limb and core strengthening in nearly half of the participants. This oversight likely contributes to the notable prevalence of lumbar pain in our study, concurring with prior research where lumbar pain was reported as the second most common complaint (15.5%) [30,37].

Interestingly, lumbar pain in our study surpasses the occurrence of lateral epicondylitis (8%), a typical overuse injury resulting from repetitive forearm movements, commonly referred to as “archer’s elbow”. This underscores the importance of core strengthening, which, coupled with lower limb conditioning, forms the foundation for supporting proper alignment. Finger injuries were reported by only 7% of our sample, differing from a study by Ertan and Tuzan (2006) that identifies fingers as the primary area of injury due to various equipment-related factors, such as the bowstring. The specialized devices used by the athletes in our study likely offered protection against such injuries, resulting in low prevalence. Nonetheless, it is essential to acknowledge equipment as a recognized extrinsic risk factor [5]. Unlike other studies, the thoracic spine region did not emerge as a frequent injury site (4.6%), and no injuries were observed in the hip region (0%) in the present research [38,39].

Our findings indicate the presence of CS symptoms in a significant proportion of young archers experiencing chronic pain, as assessed by the CSI questionnaire. Out of the 101 athletes who reported pain lasting more than three months due to training, 16 athletes (9 males and 7 females) exhibited CS symptoms. Only 32% reported a complete absence of pain or discomfort during the shot routine, while 47% faced minor, average, or major difficulties completing their usual training sessions. A concerning observation was the regular use of non-steroidal anti-inflammatory drugs before training by many young athletes, with no significantly positive effect. Additionally, some athletes reported the use of medications for depression and neuropathic pain. This finding is particularly alarming, considering the intricate interplay between chronic pain and depression [40].

Archers unable to participate in their sport due to recurrent injuries often experience depression, creating a central driver of pain [40]. Consequently, young athletes, instead of reaping the assumed health benefits from competitive archery, grapple with significant psychosocial effects and potential burnout [41]. Addressing this complex scenario requires an individually tailored multidisciplinary treatment plan that embraces a biopsychosocial approach, aiming for CS and more favorable rehabilitation outcomes [42,43].

An emphasis on competitive success has led to increased pressure to initiate high-intensity training at a young age. Under this pressure, sports medicine physicians must recognize young archers at risk for overuse injuries and provide prevention recommendations [41]. The goal should be to develop high-level young athletes with methodical efforts to ensure that the training program is sport-specific and individualized according to skeletal maturity. Continuous education in areas of archery biomechanics and increased awareness of injury prevention strategies are highly important. Coaches, sports staff, and athletes must be aware that an excessive focus on early intensive training and competition at young ages, rather than skill development, can lead to overuse injuries and burnout. Through enhanced knowledge, competitive adolescent archers can safely participate in sports and reduce the risk of repetitive injuries.

In the realm of archery, recent literature has shed light on the prevalence of both acute and overuse injuries, challenging the notion of it being a safe, non-contact sport. Factors such as training frequency, intensity, and duration, coupled with inadequate body strength and incorrect technique, contribute to the risk landscape [44]. Our study underscores the importance of controlled and guided physical training to mitigate these risks. Notably, participants in our research predominantly focused on upper body strengthening, neglecting core and lower limb conditioning, which are vital for proper alignment and stability during shooting sessions [45,46]. Proper alignment not only enhances performance but also reduces the risk of shoulder injuries [46,47,48,49], a commonly affected area in archery. Furthermore, our findings highlight the presence of chronic pain symptoms in a significant proportion of young archers, indicating the necessity for a multidisciplinary approach to address both physical and psychosocial aspects of injury prevention and rehabilitation. Through education and awareness, coaches, sports staff, and athletes can collaboratively work towards ensuring safe participation in sports while minimizing the risk of overuse injuries and burnout, thereby fostering long-term athletic sustainability and success [46].

### 4.1. Limitations

The data were collected on the day of the sports event, ensuring accurate completion of the questionnaire and a high response rate. All participating athletes completed the questionnaire on the spot, facilitating direct communication between participants and researchers regarding questionnaire consistency. However, recording the questionnaires on the same day may have influenced participant responses.

Despite being designed as a questionnaire study, our research has limitations. Firstly, recall bias may affect the reliability of retrospectively collected injury history. Additionally, the information relies on self-reports, and the definition of pain is not explicitly specified. Some adolescents may have reported muscle soreness from demanding training rather than an overuse injury. Ideally, clinical examination for each reported symptom would provide a more accurate assessment. However, conducting such examinations on the day of a sporting event is impractical. Standardized interviews followed by independent clinical examinations separate from the competition might offer more reliable results. Finally, our findings are subject to the reliability of athlete information, as some may hesitate to report symptoms, fearing the impact on their coaches’ decisions.

### 4.2. Recommendations for Future Research

Future research in the field of elite archery could benefit from longitudinal studies tracking the impact of training practices on performance and injury patterns over extended periods. Gender-specific analyses should be explored to understand differences in training practices and pain experiences. Incorporating biomechanical assessments could provide insights into optimizing archery technique and equipment to reduce musculoskeletal risks. The psychological aspects of archery training, including concentration techniques and stress management, warrant further investigation to understand their influence on pain perception and recovery. 

Moreover, research on the impact of specialized diets on performance, injury prevention programs tailored to archery, and comprehensive pain management strategies combining pharmacological and non-pharmacological approaches could contribute significantly. Integrating advanced technologies like wearable sensors for real-time monitoring of biomechanics and training loads may offer precise data on the relationship between training variables and injuries. Cultural and regional variations in archery training practices and pain experiences should also be explored. Lastly, educational programs aiming to increase awareness about balanced training, injury prevention, and effective pain management among archers, coaches, and sports professionals could be developed and evaluated for their impact.

## 5. Conclusions

This is a descriptive epidemiologic research study with the aim of quantifying injury occurrence in young archers with respect to where and when injuries occur and what their outcomes are. Our data, in addition to previously published results, should be used to reduce injury rates by driving the development of strategies to control and prevent them. Young archers report a high prevalence of injuries in the shoulder area, despite their preference for upper limb strengthening compared to other body parts. To prevent overuse injuries in archery, proper shooting technique should be achieved and muscle strengthening should also include leg and core muscles that provide body stability. Certain prevention measures should be applied so that chronic and irreversible injuries can be prevented. In such a highly demanding sport regarding concentration and technical accuracy, cognitive factors should also be addressed in addition to correcting technical adaptations. In cases of central sensitization, these two requirements are essential to progress with a successful rehabilitation program.

## Figures and Tables

**Table 1:** Descriptive characteristics of the archery athletes (N = 200).

Variables	Female Athletes *n* = 96	Male Athletes *n* = 104	Total N = 200	*p*-Value
Age (years)	16.78 (±1.67)	16.99 (±1.57)	16.89 (±0.11)	*p* = 0.36
Height (m)	1.67 (±0.07)	1.78 (±0.08)	1.73 (±0.01)	*p* < 0.01
Weight (Kg)	60.11 (±8.51)	75.60 (±13.6)	68.17 (±0.97)	*p* < 0.01
BMI	21.52 (±2.73)	23.80 (±4.81)	22.71 (±4.10)	*p* < 0.01
Training characteristics of the archery athletes (N = 200)				
Practice (years)	6.42 (±2.59)	6.64 (±3.07)	6.53 (±0.20)	*p* = 0.57
Upper arm dominance				
Right	88 (91.7%)	90 (86.5%)	178 (89%)	*p* = 0.27
Left	8 (8.3%)	14 (13.5%)	22 (11%)	*p* = 0.72
Cord pulling preference side				
Right	85 (88.5%)	92 (88.5%)	177 (88.5%)	*p* = 0.99
Left	11 (11.5%)	12 (11.5%)	23 (11.5%)	*p* = 0.99
Bow weight (lb)	15.44 (±8.01)	16.99 (±1.57)	16.89 (±1.6)	*p* = 0.33
Bow type				
Recurve	69 (71.9%)	76 (73.1%)	145 (72.5%)	*p* = 0.87
Compound	27 (28.1%)	28 (26.9%)	55 (27.5%)	*p* = 0.92
Training hours per week (h)	15.44 (±8.501)	14.38 (±7.52)	14.89 (±7.76)	*p* = 0.33
Arrows per training session	160.36 (±63.81)	165.10 (±65.07)	162.84 (±64.35)	*p* = 0.60
Number of matches	11.94 (±10.78)	10.26 (±6.92)	11.07 (±9)	*p* = 0.19
Duration of matches (min)	86.54 (±108.54)	118.72 (±127.45)	103 (±12.55)	*p* = 0.36
Training components				
Upper body strengthening	79 (82.3%)	90 (86.5%)	169 (84.5%)	*p* = 0.45
Lower body strengthening	49 (51%)	49 (47.1%)	98 (49%)	*p* = 0.70
Dominant arm strengthening	69 (71.9%)	76 (73.1%)	145 (72.5%)	*p* = 0.87
Non dominant arm strengthening	60 (62.5%)	66 (63.5%)	126 (63%)	*p* = 0.91
Aerobic conditioning	50 (52.1%)	49 (47.1%)	99 (49.5%)	*p* = 0.62
Stretching before training	73 (76%)	73 (70.2%)	146 (73%)	*p* = 0.43
Stretching after training	60 (62.5%)	56 (53.8%)	116 (58%)	*p* = 0.34
Relaxation techniques	53 (55.2%)	56 (53.8%)	109 (54.5%)	*p* = 0.87
Concentration techniques	54 (56.3%)	67 (64.4%)	121 (60.5%)	*p* = 0.37
Breathing exercises	52 (54.2%)	62 (59.6%)	114 (57%)	*p* = 0.56
Warm up	86 (92.7%)	87 (83.7%)	176 (88%)	
Duration (min)	15.01 (±8.45)	13.27 (±14.29)	14.18 (±11.595)	*p* = 0.35
Cooldown	42 (43.8%)	46 (44.2%)	88 (44%)	
Duration (min)	14.46 (±8.13)	16.12 (±18.23)	15.34 (±14.35)	*p* = 0.59
Annual physical assessment	54 (56.3%)	55 (52.9%)	109 (54.5%)	*p* = 0.72
Prescribed diet	31 (32.3%)	35 (33.7%)	66 (33%)	*p* = 0.90

**Table 2:** Mean score of CSI based on central sensitization severity.

Variables	Subclinical (CSI = 0–29) *n* = 157 78.5%	Mild (SCI = 30–39) *n* = 27 13.5%	Moderate (SCI = 40–49) *n* = 11 5.5%	Severe (SCI = 50–59) *n* = 3 1.5%	Extreme (SCI = 60–100) *n* = 2 1%	CSI Score > 40 *n* = 16 8%
CSI Score (mean ± SD)	15.37 ± 7.264	34.70 ± 2.686	42.73 ± 2.760	52.67 ± 2.887	64 ± 0	47.25 ± 8.029

**Table 3:** Correlations between CSI scores and weekly training frequency, training duration per session, number of competitions per year, and competition duration per match.

Variables	Weekly Training Frequency	Training Duration Per Session	Number of Competitions Per Year	Competition Duration Per Match
CSI Score	r = 0.20, *p* = 0.775	r = 0.69, *p* = 0.335	r = 0.33, *p* = 0.643	r = 0.08, *p* = 0.224

## Data Availability

Data are available upon reasonable request from the corresponding author.

## Supplementary Materials

The following are available online at https://www.mdpi.com/article/10.3390/sports12040101/s1, File S1: Structured questionnaires.

## Appendix A

**Table A1:** Comparative Analysis of Physical Demands, Injury Risks, and Training Characteristics in Archery and Other Non-Contact Sports.

Aspect	Archery [22,50]	Swimming [51,52]	Tennis [53,54]	Golf [55,56]
**Physical Demands**	Requires upper body strength and stability, with emphasis on shoulder and back muscles.	Emphasizes full-body aerobic endurance, along with upper body and core strength.	Demands agility, speed, and coordination, with rapid changes in direction and pace.	Focuses on precision and control, with minimal physical exertion.
**Injury Risk**	Risk of overuse injuries in the shoulder and back due to repetitive movements.	Low risk of overuse injuries, but potential for shoulder injuries from improper technique.	Moderate risk of overuse injuries due to repetitive movements and intense play.	Lower risk of overuse injuries, but potential for strain from swinging motion.
**Mental Focus**	Requires intense concentration and mental discipline to maintain consistent form and accuracy.	Demands focus on technique and pacing, with attention to breathing and stroke mechanics.	Requires mental agility, quick decision-making, and adaptability during matches.	Emphasizes mental toughness and concentration, especially during precision shots.
**Training Intensity**	Training involves regular practice to build muscle memory and refine technique, with occasional breaks	Involves structured workouts and interval training to build endurance and improve technique.	Includes drills, matches, and physical conditioning to improve endurance and skill.	Practice typically involves repetitive swinging motions and putting techniques.
**Equipment**	Requires specialized equipment including bows, arrows, and protective gear.	Requires minimal equipment, with focus on swimwear and goggles.	Requires specific gear like rackets, balls, and appropriate footwear for surface.	Requires clubs, balls, and tees, but less frequent equipment purchases.
