# Supplementary material for: Injuries and Persistent Pain in Elite Adolescent Archery Athletes: A Cross-Sectional Epidemiological Study
Source: Sports (Basel). 2024 Apr 2;12(4):101. doi: 10.3390/sports12040101 (PMC11054429; doi:10.3390/sports12040101)
Supplement: Supplementary file 1 [file sports-12-00101-s001.zip › sports-2898758-supplementary.pdf]

1 Personal Athlete Information

Athlete Identification Number:___________________________________

Nationality:__________________________________

Age:_____

Gender: Female □ Male□

Years of Practicing With Archery:_____________________

Height:__________ Weight:___________

Hand you write? Right □ Left □

Hand you pull the cord; Right □ Left □

2 Training/Bow

1. How many libres weight the bow you use? ___________________

2. What type of bow do you use? Recurve □ Compound □

3. How many days did you train in the most recent (In the last) season?_____________

4. How long (in minutes) did each training session last in the most recent season?

5. On average, how many arrows do you shoot in each practice? _____________

6. How many matches have you competed in this most recent season?____________________________

7. On average, how long (in minutes) does each match last?_____________

8. Which of the below are part of your training routine?

Upper Body Strength training	Yes □
Lower Body Strength training	Yes □
Right arm strength training	Yes □
Left arm strength training	Yes □
Aerobic conditioning	Yes □
Stretches at the beginning of training	Yes □
Stretches at the end of training	Yes □
Relaxation techniques	Yes □
Concentration techniques	Yes □
Breathing exercises	Yes □
Other	

9. Do you warm up before each training session? Yes □ No □

10. If so, how many minutes? _______________

11. Do you perform a cooldown after each training session? Yes □ No □

12. If so, how many minutes? _______________

13. During the year, do you undergo physical evaluation (join range of motion, muscular strength)? Yes □ No □

14. Do you have a specific nutritional plan; Yes □ No □

3. Training related pain

1. Do you experience any persistent pain for a long period of time that is due to training? Yes □ No □

2. If you have a pain or discomfort during training, do you tell to your coach? Yes □ No □

3. If you have a pain, do you take any medication for it so you can train? Yes □ No □

4. Do you take any medication regularly?

____

5. Does the medication you take help with the pain or discomfort? None □, Slight □, Some □, Much □, Very Much □

6. If you experience any chronic pain during training, fill in the chart below (one chart for each site of pain)

Front

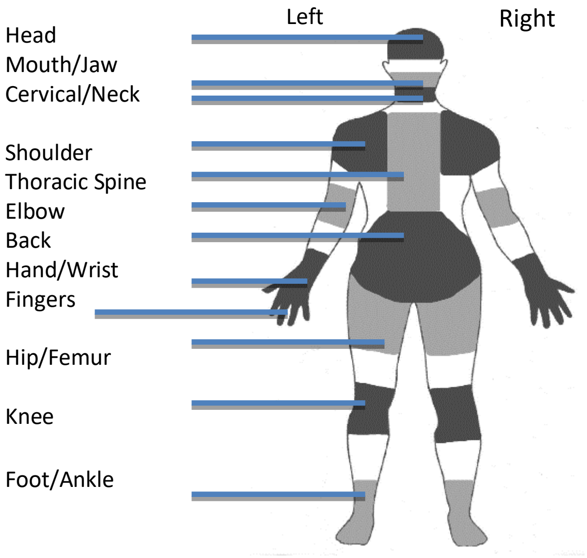

Back

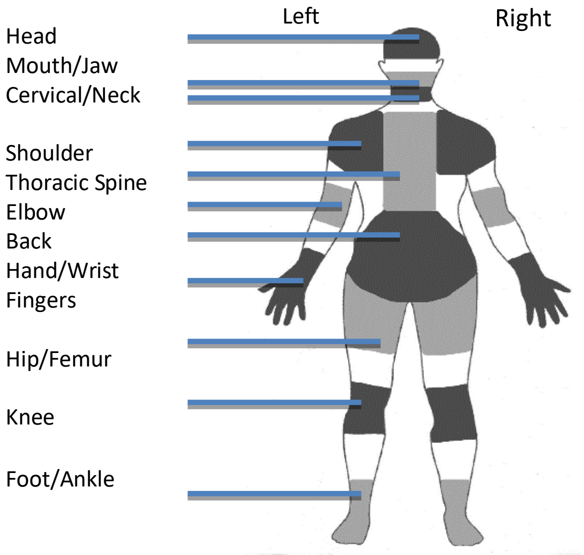

Pain number…

**Did an injury occur?**	**How long ago did the pain start?** (days/moment)	**Pain duration;** (days/months)	**Type of pain** (Numbness, sharp, burning sensation, dull, pinching, other)	**Usual pain intensity;** (0= no pain, 10 = extreme pain)	**Diagnosis;** (ex; tendinitis, back pain, rotator cuff, etc.)	**Did you have to stop training;**	**For how long were you unable to train?** (days)	**Did you have physical therapy?**	**How many sessions?** (number)	**Was any improvement ?**
Yes□ No □				/10		Yes □ No □		Yes □ No □		None □<br>Slight □<br>Some □<br>A lot of □<br>Very Much □

Front **Back**

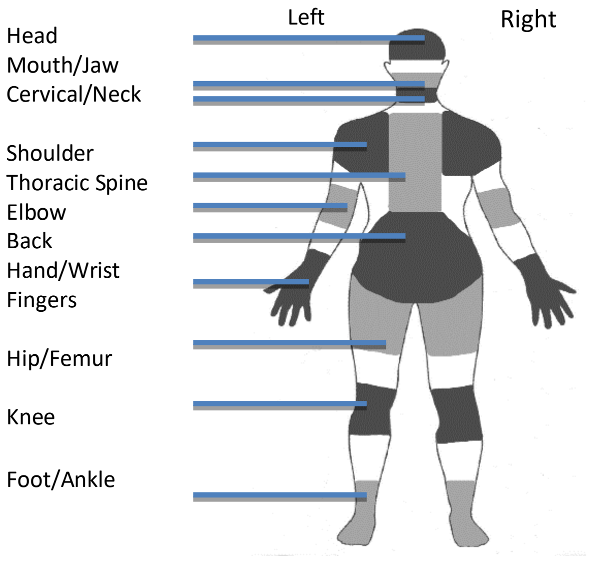

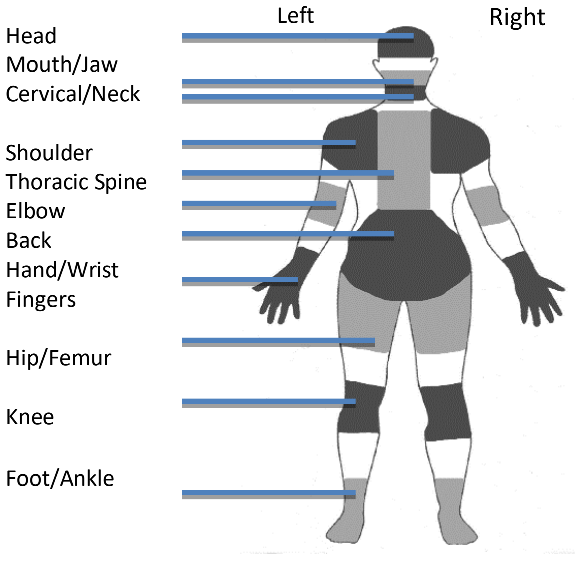

Pain number…

**Did an injury occur?**	**How long ago did the pain start?** (days/moment)	**Pain duration;** (days/months)	**Type of pain** (Numbness, sharp, burning sensation, dull, pinching, other)	**Usual pain intensity;** (0= no pain, 10 = extreme pain)	**Diagnosis;** (ex; tendinitis, back pain, rotator cuff, etc.)	**Did you have to stop training;**	**For how long were you unable to train?** (days)	**Did you have physical therapy?**	**How many sessions?** (number)	**Was any improvement ?**
Yes☐ No ☐				/10		Yes ☐ No ☐		Yes ☐ No ☐		None ☐<br>Slight ☐<br>Some ☐<br>A lot of ☐<br>Very Much ☐

Front

Head
Mouth/Jaw
Cervical/Neck

Shoulder
Thoracic Spine
Elbow
Back
Hand/Wrist
Fingers

Hip/Femur

Knee

Foot/Ankle

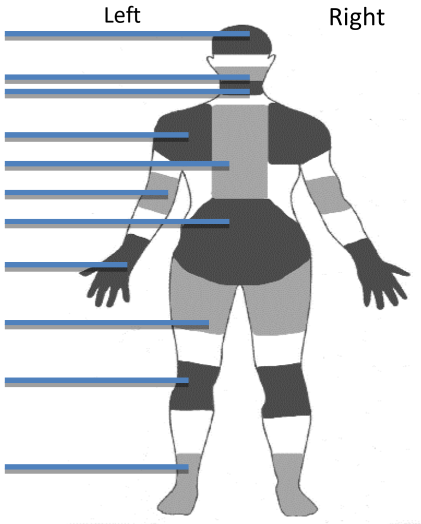

Back

Head
Mouth/Jaw
Cervical/Neck

Shoulder
Thoracic Spine
Elbow
Back
Hand/Wrist
Fingers

Hip/Femur

Knee

Foot/Ankle

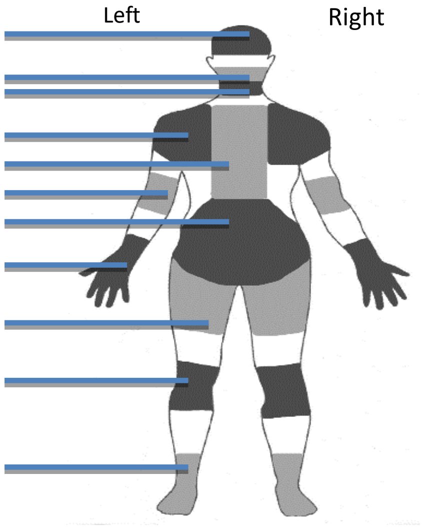

Pain number…

**Did an injury occur?**	**How long ago did the pain start?** (days/moment)	**Pain duration;** (days/months)	**Type of pain** (Numbness, sharp, burning sensation, dull, pinching, other)	**Usual pain intensity;** (0= no pain, 10 = extreme pain)	**Diagnosis;** (ex; tendinitis, back pain, rotator cuff, etc.)	**Did you have to stop training;**	**For how long were you unable to train?** (days)	**Did you have physical therapy?**	**How many sessions?** (number)	**Was any improvement ?**
Yes☐ No ☐				/10		Yes ☐ No ☐		Yes ☐ No ☐		None ☐<br>Slight ☐<br>Some ☐<br>A lot of ☐<br>Very Much ☐

7. Please circle the appropriate answer which accurately describes your physical condition **during the last week**

	No difficulty	Mild difficulty	Moderate difficulty	Severe difficulty	Unable
**Did you have any difficulty using your usual technique with your bow?**	1	2	3	4	5
**Did you have any difficulty training due to any pain?**	1	2	3	4	5
**Did you experience any difficulty shooting in training as well as you would like?**	1	2	3	4	5
**Did you have any difficulty spending your usual amount of time training?**	1	2	3	4	5

4. General Health Status

1. Have you had any medical procedures performed in the past? (ex, surgery)

____

____

2. Do you have any other general medical conditions? (ex, high blood pressure, diabetes, thyroidism, etc.)

____

____

This is the last question. Thank you for your cooperation!
